# Hearing impairment in Stickler syndrome: a systematic review

**DOI:** 10.1186/1750-1172-7-84

**Published:** 2012-10-30

**Authors:** Frederic R E Acke, Ingeborg J M Dhooge, Fransiska Malfait, Els M R De Leenheer

**Affiliations:** 1 Department of Otorhinolaryngology, 1P1, Ghent University / Ghent University Hospital, De Pintelaan 185, Ghent 9000, Belgium; 2 Department of Medical Genetics, Ghent University Hospital, De Pintelaan 185, Ghent, 9000, Belgium

**Keywords:** Stickler syndrome, Arthro-ophthalmopathy, Collagen, *COL2A1*, Hearing loss, Cleft palate

## Abstract

**Background:**

Stickler syndrome is a connective tissue disorder characterized by ocular, skeletal, orofacial and auditory defects. It is caused by mutations in different collagen genes, namely *COL2A1*, *COL11A1* and *COL11A2* (autosomal dominant inheritance), and *COL9A1* and *COL9A2* (autosomal recessive inheritance). The auditory phenotype in Stickler syndrome is inconsistently reported. Therefore we performed a systematic review of the literature to give an up-to-date overview of hearing loss in Stickler syndrome, and correlated it with the genotype.

**Methods:**

English-language literature was reviewed through searches of PubMed and Web of Science, in order to find relevant articles describing auditory features in Stickler patients, along with genotype. Prevalences of hearing loss are calculated and correlated with the different affected genes and type of mutation.

**Results:**

313 patients (102 families) individually described in 46 articles were included. Hearing loss was found in 62.9%, mostly mild to moderate when reported. Hearing impairment was predominantly sensorineural (67.8%). Conductive (14.1%) and mixed (18.1%) hearing loss was primarily found in young patients or patients with a palatal defect. Overall, mutations in *COL11A1* (82.5%) and *COL11A2* (94.1%) seem to be more frequently associated with hearing impairment than mutations in *COL2A1* (52.2%).

**Conclusions:**

Hearing impairment in patients with Stickler syndrome is common. Sensorineural hearing loss predominates, but also conductive hearing loss, especially in children and patients with a palatal defect, may occur. The distinct disease-causing collagen genes are associated with a different prevalence of hearing impairment, but still large phenotypic variation exists. Regular auditory follow-up is strongly advised, particularly because many Stickler patients are visually impaired.

## Introduction

Stickler syndrome or hereditary progressive arthro-ophthalmopathy [1] (ORPHA828) is a connective tissue disorder affecting about 1/7,500 to 1/9,000 newborns [2]. It is characterized by ocular, skeletal, orofacial, and auditory abnormalities. Typical features include vitreoretinal degeneration, high-grade myopia, retinal detachment, premature osteoarthritis, midfacial hypoplasia, cleft palate and hearing loss [3,4].

Stickler syndrome is subdivided into several subtypes, based on its underlying genetic collagen defect. At present, defects in three different collagen genes have been found in patients with autosomal dominant Stickler syndrome. Type I Stickler syndrome (STL1) is associated with mutations in the *COL2A1* gene encoding type II collagen [5], while mutations in *COL11A1* and *COL11A2* encoding type XI collagen, are associated with type II (STL2) [6] and type III Stickler syndrome (STL3) [7] respectively. Autosomal recessive Stickler syndrome has been described in some families with mutations in *COL9A1* (STL4) [8] and *COL9A2* (STL5) [9] encoding type IX collagen.

Phenotypic distinction between patients with mutations in different causative genes is possible to a certain degree. For example, STL3 does not exhibit ocular abnormalities, as *COL11A2* is not expressed in the vitreous [10]. Another example is the vitreous anomaly, which is mostly ‘membranous’ in STL1 and ‘beaded’ in STL2 [6]. However, large phenotypic difference, not explained solely by the affected gene, still exists. Even within one family or within unrelated families carrying the same mutation, clinical expression shows high variability [11].

In 1965, Stickler et al. described a family with joint manifestations and progressive myopia, associated with retinal detachment in the first decade of life and resulting in blindness [1]. In an additional report, mild sensorineural hearing impairment was added to the features typical of this syndrome [12]. Subsequently, hearing impairment has commonly been reported as a symptom of Stickler syndrome, though detailed descriptions are scarce and few studies are primarily focused on the auditory phenotype. Hearing loss in STL1 seems to be present in about 60% of affected persons and is likely to be sensorineural [13,14]. STL2 and STL3 are associated with hearing loss occurring more frequently and being more severe [13]. The pathogenesis of this sensorineural hearing loss is not well understood. Associated findings are a hypermobile tympanic membrane [14], and cleft palate resulting in middle ear effusion and conductive hearing loss.

Because of the large interfamilial and intrafamilial phenotypic variability observed in Stickler patients, clinicians are unfortunately unable to predict whether their patients will develop hearing loss. In this article, we aim to review the literature about hearing impairment in all types of Stickler syndrome. The auditory features of patients found by systematic review is described and prevalences, obtained through meta-analysis, are provided. Hearing loss is also linked to the causal gene, mutation type and mutation effect in order to find correlations. This article offers an up-to-date overview of hearing loss in Stickler syndrome, correlated with the genotype.

## Materials and methods

### Search strategy

A systematic review was performed in order to analyze the auditory features in Stickler syndrome. We intended to find all papers describing the phenotype with regard to hearing in Stickler patients, along with their genotype.

Relevant articles were searched using the electronic databases of PubMed and Web of Science from inception to 31 July 2012. Considering the different notations (e.g. Stickler syndrome vs. Stickler’s syndrome), following search strings were used: “stickler*” and “arthro-ophthalmopath* OR arthroophthalmopath*”. To exclude non-relevant articles, the queries were restricted to title/abstract in Pubmed and topic in Web of Science. Reference lists of the retrieved articles were hand-searched for additional papers. A bibliography with the citations retrieved from the abovementioned searches was created using EndNote X4 (Thomson Reuters, New York, USA). Duplicates and non-English articles were removed both automatically and manually, as well as short conference proceedings. One investigator (FA) conducted this first selection of articles, under the auspices of the principal investigators (EDL, ID), in order to find articles for review purposes.

To perform a meta-analysis, the eligibility of papers was assessed based on two main inclusion criteria: the indication of the presence or absence of hearing impairment in Stickler and Stickler-like patients and the finding of a causative mutation. Probable Marshall phenotype and autosomal recessive otospondylomegaepiphyseal dysplasia (OSMED) patients were excluded, as well as patients in whom only linkage to a gene was demonstrated. In case of doubt, mutual agreement between the authors was achieved.

### Statistical analysis

The following data, if available, were extracted from articles meeting the inclusion criteria: study characteristics (authors, year of publication, study design and methods, original data or described elsewhere), patient attributes (family and age, relevant comorbidities), hearing features (hearing impairment, type and severity of hearing loss, additional auditory data, palatal defects) and mutation (mutated gene, mutation type and location, mutation effect). When a patient or family was described in different papers, the most informative paper was used for data collection.

Calculations were performed using SPSS version 19 (SPSS Inc., Chicago, USA). Where appropriate, statistical tests were used to assess statistical significance. Exons of *COL2A1* were numbered according to the GenBank database [GenBank:L10347]. In order to avoid selection bias (proportionally greater importance of larger families), some calculations were repeated in an analysis where all families were equally weighted. In the subtypes of Stickler syndrome where sufficient audiograms of different patients could be collected, an average audiogram or even an age-related typical audiogram (ARTA) of the respective subtype was calculated.

The study was conducted taking into account the instructions of the PRISMA statement for reporting systematic reviews [15] and of the MOOSE group for reporting a meta-analysis of observational studies [16].

## Results

### Search results

**Figure 1:**
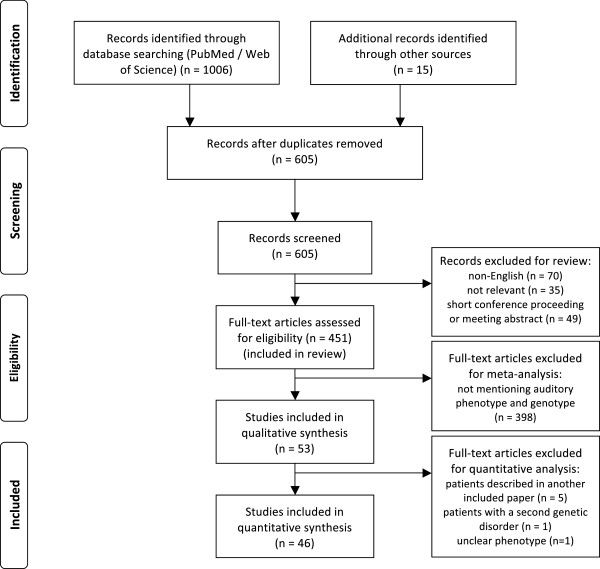
**PRISMA flow diagram, showing the overview of the search process.** Schematic overview of the various steps involved in the search process, according to the PRISMA guidelines. We read 451 articles about Stickler syndrome, whose content, if relevant, could be included in the discussion. Data from 46 articles, meeting the inclusion criteria of the meta-analysis, are used to provide accurate prevalences about hearing loss in the results section of this article.

A flow diagram of the search process is depicted in Figure 1. For review purposes, 451 articles were read to extract interesting data described in the discussion. Ultimately, 46 articles were included in the meta-analysis (see Additional file 1: Table S1 for description of the included articles) [3,5,8,9,11,17-57]. Seven papers that fulfilled the inclusion criteria were withheld because of different reasons. Five of them contained data of patients being described more thoroughly in another paper [6,7,58-60], one reported a patient suffering from a second genetic disorder that could as well result in hearing loss [61], and hearing impairment in another one was unclear [62]. Quality of the included papers was assessed (Additional file 1: Table S1), but none of them were rejected based on quality properties alone, in order to obtain a large population in which it is possible to draw firm conclusions. In the 46 included articles, a total of 313 individual patients of 102 families were found to meet the inclusion criteria. Nine additional families with phenotypic and genotypic information, but without individual data, were added, resulting in 111 different Stickler families.

### Auditory phenotype in Stickler syndrome

Mean age of the individual Stickler patients was 26.4 years (standard deviation 19.49, age was given in 221 patients), and gender distribution was 55.2% female versus 44.8% male persons (gender was provided in 210 patients).

**Figure 2:**
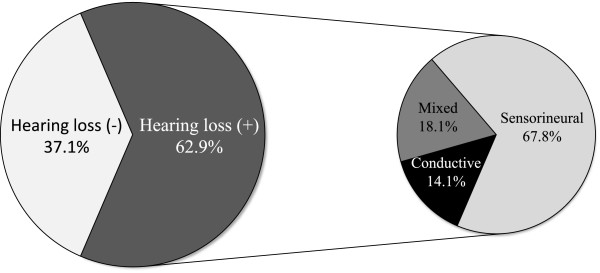
**The prevalence and type of hearing loss in Stickler syndrome.** The percentages of the 313 included Stickler patients without and with hearing loss are displayed. Of the hearing-impaired patients, a subdivision into conductive, mixed and sensorineural hearing loss is provided. The group of hearing-impaired patients in which the type was not mentioned (6.4% of the total population), was proportionally divided among the three hearing loss groups.

**Figure 3:**
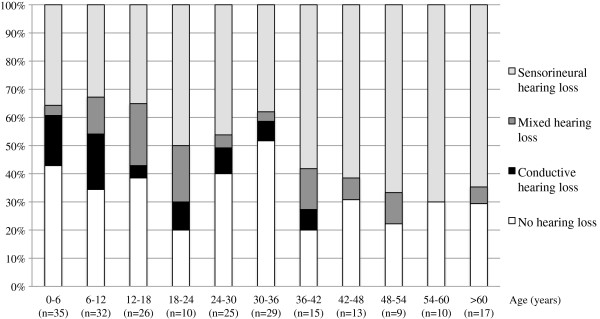
**Prevalences of hearing phenotype in Stickler patients, divided into age groups.** The percentages of hearing loss and its type for each age group can be derived from this diagram. The group of hearing-impaired patients in which the type was not mentioned (6.4%), was proportionally divided among the three hearing loss groups.

Hearing loss was reported in 197 of the 313 patients (62.9%, Figure 2). In 177 cases, the type of hearing loss was mentioned. In this group 120 patients (67.8%) showed a pure sensorineural hearing loss. Mixed hearing loss was present in 32 persons (18.1%), and 25 patients (14.1%) suffered from conductive hearing loss. Consequently, a conductive component (conductive and mixed hearing loss) was present in 32.2% of the hearing-impaired patients. Figure 3 shows the distribution of prevalence and type of hearing loss among different age groups (age was available in 221 patients). When splitting up the hearing-impaired population in which age is available into a group of children (below 18 years) and a group of adults (aged 18 and older), hearing loss included a conductive component in 46.6% (27/58) in the younger group versus 23.3% (20/86) in the older group (p = 0.004, Fisher exact test). Gender did not significantly alter the prevalence of hearing loss, not even at high age (p = 0.31 for the whole group, Fisher exact test). Regarding severity, hearing loss was mild in 44.6%, moderate in 36.6% and severe in 18.8% of the subjects, although it is often not clear which definitions for severity were applied. No profound hearing loss was observed. In patients with mixed hearing loss, severity was often higher; however, a conductive component can only be moderate at worst.

A palatal defect (cleft palate, submucous cleft, high-arched palate) was present in 42.6% (121/284) of the studied population. Of the patients with a palatal defect, 73.6% (89/121) were hearing-impaired, compared to 54.0% (88/163) without palatal defect (p < 0.001, Fisher exact test). As seen in Table 1, this difference can largely be attributed to the higher prevalence of conductive and mixed hearing loss, while the percentage of sensorineural hearing loss is comparable between the two groups.

**Table 1:** Prevalence and type of hearing loss in patients with and without palatal defect

	**Palatal defect (n = 121)**	**No palatal defect (n = 163)**	**p value**
**No hearing loss**	26.4%	46.0%	***<0.001***
**Conductive hearing loss**	13.6%	4.2%	***0.008***
**Mixed hearing loss**	20.0%	6.3%	***<0.001***
**Sensorineural hearing loss**	40.0%	43.5%	0.544

This table shows the percentages of each type of hearing phenotype for patients with and without palatal defect separately. Palatal defects include cleft palate, submucous cleft and high-arched palate. The group of hearing-impaired patients in which the type was not mentioned, was proportionally divided among the three hearing loss groups. Stickler patients with a palatal defect exhibit significantly more hearing loss, and this can be attributed to the higher number of patients with conductive and mixed hearing loss.

### Auditory phenotype in different Stickler genotypes

**Figure 4:**
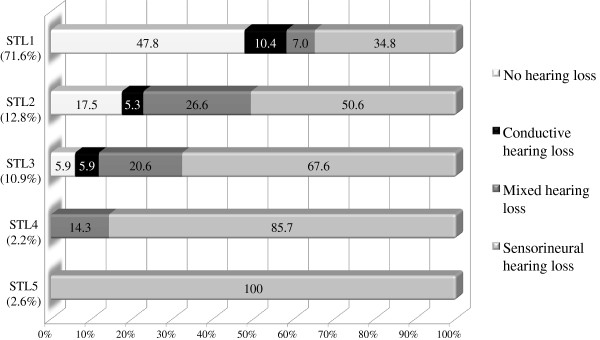
**Hearing phenotype in distinct Stickler types.** The percentages of hearing loss and its type for each type of Stickler syndrome (according to the affected gene) are shown in this figure. The group of hearing-impaired patients in which the type was not mentioned (6.4%), was proportionally divided among the three hearing loss groups.

**Figure 5:**
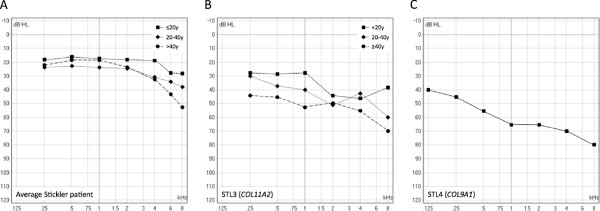
**Typical audiograms for Stickler syndrome in general, and for STL3 and STL4. A**) ARTA for Stickler patients in general (without genetic subclassification), based on the detailed audiometric results in 44 patients [14]. Mean air conduction thresholds of both ears are shown. **B**) ARTA for type III Stickler syndrome, based on the detailed audiometric results in 18 STL3 patients (52 different measurements) [17,53]. Air conduction thresholds of the best ear are shown, unless in patients where an air-bone gap was present, in which bone conduction of the best ear is shown. **C**) Average audiogram for type IV Stickler syndrome, based on the detailed audiometric results in 7 STL4 patients (median age 15y) [8,41]. Air conduction thresholds of the best ear are shown.

STL1 occurred in 224 cases (71.6%), while this was 40 (12.8%) for STL2, 34 (10.9%) for STL3, 7 (2.2%) for STL4 and 8 (2.6%) for STL5. For each Stickler gene, the distinct percentage and type of hearing loss is shown in Figure 4. Severity of hearing loss was mostly reported to be mild to moderate for STL1, STL2 and STL5. In STL3 and STL4, predominantly moderate and severe hearing loss was found respectively. An ARTA for STL3 and an average audiogram for STL4 are provided in Figure 5, as well as an ARTA for Stickler syndrome in general. Palatal defects were present in 44.3%, 48.7% and 44.8% in STL1, STL2 and STL3, respectively. Patients with a collagen IX mutation (STL4 and STL5) did not exhibit palatal defects.

The mutations of 36 STL1 patients were expected to result in a dominant-negative effect, while these of 174 STL1 patients were supposed to induce nonsense-mediated decay (mutation effect was available in 210 STL1 patients). Hearing loss was present in 47.2% of the first group versus 52.9% of the second (p = 0.59, Fisher exact test), and a palatal defect occurred in 16.7% versus 46.9% (p = 0.002, Fisher exact test). No statistical significance was observed between location of the mutation in the collagen gene (exon/intron number) and the presence of hearing loss (p = 0.44, Mann–Whitney U test; mean exon number of 26.0 when hearing loss is absent vs. 28.8 when present), but differences were seen between mutation location and palatal defects (p = 0.01, Mann–Whitney U test; mean exon number of 24.4 when palatal defect is absent vs. 30.4 when present).

### Auditory phenotype and genotype in family study

When the 111 different families are equally weighted, regardless of the number of affected family members, the prevalence of hearing loss was 60.1%. Within these hearing-impaired subjects, the percentages of sensorineural, mixed and conductive hearing loss were 60.4%, 18.3% and 21.3%, respectively. The prevalence of palatal defects was 51.5%. STL1 occurred in 85 families (76.6%), while this was 17 (15.3%) for STL2, 5 (4.5%) for STL3, 3 (2.7%) for STL4 and 1 (0.9%) for STL5.

## Discussion

Hearing loss is a common feature in Stickler syndrome, expressed in 63% of all patients. About two-thirds of these hearing-impaired patients express a purely sensorineural loss. This prevalence increases with advancing age, most probably not only due to age-related hearing loss. Conductive and mixed hearing loss is mostly found in children with Stickler syndrome and in patients with a history of a palatal defect, but can also be present in adults. These results are consistent with those of other authors studying hearing features in a large group of Stickler patients [14,63]. As the distinct disease-causing collagen genes in Stickler syndrome lead to a different auditory phenotype, hearing features are described for the involved genes separately.

About 75% of all Stickler patients have type I Stickler syndrome, caused by mutations in *COL2A1*. This type has the best prognosis concerning hearing: 52.2% of the affected persons exhibit hearing loss, which is mostly sensorineural. As in the other dominant Stickler types, conductive and mixed hearing loss may also be present, especially in young children and with a palatal defect. The sensorineural hearing loss is often reported to affect mainly the higher frequencies. Progression of hearing impairment is described, but it is unclear whether the high-frequency hearing loss in STL1 progresses beyond presbyacusis. It is generally mild to moderate and does not evolve to severe hearing impairment.

*COL2A1* is present in numerous inner ear structures, which may explain why mutations in this gene can cause hearing loss [64]. Stickler syndrome is just one of the clinical expressions that belongs to the spectrum of type II collagenopathies, ranging from the lethal achondrogenesis type II, to mild forms of solely ocular or skeletal manifestations. Some of these expressions are also associated with hearing loss. However, large variability, interfamilial as well as intrafamilial, exists. Our study failed to find significant differences in STL1 hearing loss due to mutation effect and location. Therefore, it can be suggested that modifier genes and environmental factors may also play a role in the development of hearing loss.

Type II Stickler syndrome (*COL11A1*) is the second most common type of Stickler syndrome. It is estimated to be present in about 15% of the probands with Stickler syndrome. Hearing loss is found in 82.5% of these patients; most of them have sensorineural loss, but again, also conductive and mixed hearing impairment have been described. Hearing loss in STL2 seems to be more pronounced than in STL1, and is already apparent at young age. It is not clear whether the higher frequencies are predominantly affected and whether or not the hearing loss progresses with age.

Marshall syndrome is allelic with STL2 and both diseases show considerable overlap. Most of the Marshall patients also develop early-onset, high-frequency sensorineural hearing loss progressing to severe hearing impairment at older ages.

Type XI collagen is associated with type II collagen and hybridization of their gene products can be found in the lateral wall of the developing mouse cochlea. It is suggested that mutations in *COL11A1* may influence hearing due to their effects on the formation and function of the tectorial membrane [65]. Until now, it is not yet clarified how mutations in this gene can cause the above-mentioned type of hearing loss. However, although not quantitatively important in the inner ear, *COL11A1* protein seems to be crucial for normal hearing.

In type III Stickler syndrome (*COL11A2*), hearing loss was found in 94.1% of the patients. It can even be suggested that all STL3 patients have some degree of hearing loss, because the two subjects not exhibiting hearing problems, were not audiometrically tested [51]. Sensorineural hearing impairment is, as in STL1 and STL2, most prevalent, and seems to be more pronounced in the middle and higher frequencies. However, there is no typical audiometric configuration and a conductive component may also be present [17]. Hearing loss in STL3 is likely to have a childhood onset and does not or just slightly progress; its severity is mostly moderate.

Mutations in *COL11A2* may also result in non-syndromic hearing loss as in DFNA13 and DFNB53. Hearing impairment in DFNA13 is non-progressive, probably prelingual, and represented by a U-shaped to slightly down-sloping audiogram [66]. Interestingly, one family with DFNA13 seemed to be protected against presbyacusis [67], which might also be present in type III Stickler syndrome [53]. DFNB53-related hearing impairment results in a prelingual, severe, U-shaped audiogram [68]. Autosomal recessive OSMED is caused by mutations in *COL11A2* as well. This syndrome is characterized by severe sensorineural hearing loss, enlarged epiphyses, shortness of the limbs and orofacial features comparable to those of Stickler syndrome [69].

Previous studies revealed the disruption of collagen fibrils within the tectorial membrane to be the only observed morphological inner ear change of *COL11A2* −/− mice, resulting in a frequency-independent, cochlear loss of 30-50 dB [66,70]. Consequently, collagen type XI is important to maintain the tectorial membrane integrity, as both *COL11A1* and *COL11A2* mutations may cause hearing loss due to changed morphology of this inner ear structure.

Four different families with autosomal recessive Stickler syndrome have been described, all exhibiting a mutation in genes encoding type IX collagen [8,9,41]. The involved patients showed a similar pattern of hearing impairment: a slightly progressive sensorineural hearing loss with early onset, more pronounced at higher frequencies. Severity was moderate to severe in STL4 (*COL9A1*) and mild to moderate in STL5 (*COL9A2*). None of these patients showed palatal defects.

These results are consistent with animal studies, which demonstrated type IX collagen, in addition to type XI collagen, as an important factor in maintaining the integrity of collagen fibers in the tectorial membrane [71]. Type IX collagen knock-out mice showed progressive hearing loss and morphological changes of the tectorial membrane, starting in the basal turn of the cochlea and progressing towards the apical turn [72]. This is in line with the observed hearing loss in recessive Stickler syndrome: progressive and initially more pronounced at higher frequencies.

Besides Stickler syndrome, mutated type IX collagen can also result, inter alia, in multiple epiphyseal dysplasia (MED), a skeletal dysplasia not associated with hearing loss. However, MED has been attributed to heterozygous splice site mutations in type IX collagen genes, resulting in in-frame exon skipping situated in the third collagenous domain (COL3) and effectuating a dominant-negative effect [41,73]. In contrast, the mutations found in STL4 and STL5 are more spread out over the gene and are supposed to result in nonsense-mediated decay.

Another, rather specific, auditory finding in Stickler syndrome is hypermobility of the tympanic membrane, measured by tympanometry. In up to 46% of Stickler patients, type A_D_ tympanograms were obtained [3,11,14]. Most of these patients probably had STL1, because tympanic hypermobility was less seen in STL2 patients [74]. Hypermobile middle ear system may be the result of frequent otitis media with ventilation tubes and/or tympanic perforations, but the collagen defect in STL1 may also contribute. Indeed, type II collagen is observed in the ossicular joints [75] and is the most abundant collagen of the tympanic lamina propria [76].

In Stickler patients, language and speech may be affected by hearing impairment and by the typical facial morphology, including cleft palate, especially if these problems are not recognized early and optimally treated. However, speech audiometry showed perception and discrimination scores consistent with the obtained pure tone thresholds [17].

Little attention is paid to the vestibular system of Stickler patients. Ocular and skeletal manifestations can contribute to instability complaints and may interfere with some of the balance tests [14]. Five tested children and 1 adult have been identified as having abnormal peripheral vestibular function combined with hearing loss [77]. In their review, Admiraal et al. stated that Stickler patients rarely complain about vestibular symptoms, though vestibular deficits may be objectified [13]. Future research should also focus on the vestibular system, as this may render novel insight into the pathophysiology of the syndrome.

Temporal bone imaging in Stickler syndrome mostly does not reveal middle or inner ear structural anomalies (5 cases mentioned by Rai et al. [78], 19 cases in Szymko-Bennett et al. [14]). In sporadic cases, ossicular defects [4] and fixations [21] were reported. Again, more studies need to be performed to draw strong conclusions about temporal bone anomalies in Stickler syndrome.

Conductive hearing loss in adults is sporadically reported and can be due to stapes ankylosis [21], chronic ear disease resulting in mastoidectomy surgery [17,43], and eustachian tube dysfunction, predominantly in patients with a history of cleft palate. With the latter in mind, clinicians should also be aware of cholesteatoma development in Stickler patients.

Overall, when interpreting these results, some comments have to be made. A family analysis was performed in order to avoid bias due to the greater importance of families in which a large number of affected family members are described. When the different families are equally weighted, only minor differences could be found concerning the auditory phenotype. To avoid reporting bias, we also included papers in which hearing features were not extensively described or studied. However, quality analysis of the studied articles revealed that hearing loss seems to be found more in studies with auditory testing compared to history alone. Consequently, we confirm that clinicians cannot rely on patients’ history of hearing capacity alone, and that regular hearing tests in Stickler patients are recommended, regardless of symptom reporting [79]. Future research should focus on progression of hearing loss and the audiometric configuration, especially the presence and practical influence of high-frequency sensorineural loss. These results may even contribute in unravelling the pathophysiology of presbyacusis. Novel Stickler studies should take into account age-related hearing loss, as few studies to date did, and a uniform classification system should be used. It would also be interesting to study other relevant topics, such as imaging of the middle and inner ear structures, and functioning of the vestibular system, as these are not well described in literature.

## Conclusions

Hearing loss is a common finding in Stickler syndrome, affecting more than half of the patients. Sensorineural hearing loss predominates, but conductive and mixed hearing loss may also be present, especially in young children and in patients with an associated palatal defect. There are differences in prevalence of hearing loss for the different types of Stickler syndrome and each genotype shows a different pattern of hearing loss. Hearing loss in STL1 is present in half of the patients and is usually mild to moderate, while hearing impairment in STL2 and STL3 is more common, more severe, and present at younger age. However, we have to be aware of large phenotypic variation. Given the high prevalence of hearing loss in Stickler patients, referral for hearing assessment and regular auditory follow-up is required upon diagnosis. If hearing impairment is detected, site of lesion testing will guide treatment options, such as ventilation tube insertion and hearing aid fitting. Close attention to the hearing system is particularly important because most of the patients are already visually impaired.

## Abbreviations

STL1: Stickler syndrome type I (*COL2A1*); STL2: Stickler syndrome type II (*COL11A1*); STL3: Stickler syndrome type III (*COL11A2*); STL4: Stickler syndrome type IV (*COL9A1*); STL5: Stickler syndrome type V (*COL9A2*); OSMED: Otospondylomegaepiphyseal dysplasia; ARTA: Age-related typical audiogram; DFNA: Deafness, autosomal dominant; DFNB: Deafness, autosomal recessive; MED: Multiple epiphyseal dysplasia.

## Competing interests

The authors declare that they have no competing interests.

## Authors’ contributions

FA collected the data, performed the statistical analysis and drafted the manuscript. ID and EDL participated in the design of the study, reviewed the data collection independently and made contributions to the draft of the manuscript. FM was involved in genetic data interpretation and in drafting the manuscript. All authors read and approved the final manuscript.

## Supplementary Material

Additional file 1 **Table S1. **List of articles included in the meta-analysis. This table shows the articles from which data were extracted to use in the results section. For each article, the number of patients and families included, and the type of Stickler syndrome is mentioned, as well as some methodological features. References are numbered according to and can be found in the original article. Click here for file
