# Supplementary material for: Hearing impairment in Stickler syndrome: a systematic review
Source: Orphanet J Rare Dis. 2012 Oct 30;7:84. doi: 10.1186/1750-1172-7-84 (PMC3551705; doi:10.1186/1750-1172-7-84)
Supplement: Additional file 1 — Table S1. List of articles included in the meta-analysis. This table shows the articles from which data were extracted to use in the results section. For each article, the number of patients and families included, and the type of Stickler syndrome is mentioned, as well as some methodological features. References are numbered according to and can be found in the original article. [file 1750-1172-7-84-S1.docx]

**Hearing impairment in Stickler syndrome: a systematic review**

***Additional file***

Authors:

Frederic RE Acke^1*^, Ingeborg JM Dhooge^1^, Fransiska Malfait^2^, Els MR De Leenheer^1^

^1^Department of Otorhinolaryngology, Ghent University Hospital, De Pintelaan 185, 9000 Ghent, Belgium

^2^Department of Medical Genetics, Ghent University Hospital, De Pintelaan 185, 9000 Ghent, Belgium

Email: Frederic Acke [frederic.acke@ugent.be](mailto:frederic.acke@ugent.be), Ingeborg Dhooge [ingeborg.dhooge@ugent.be](mailto:ingeborg.dhooge@ugent.be), Fransiska Malfait [fransiska.malfait@ugent.be](mailto:fransiska.malfait@ugent.be), Els MR De Leenheer [els.deleenheer@ugent.be](mailto:els.deleenheer@ugent.be)

^*^Corresponding author:

Frederic Acke, MD

Ghent University / Ghent University Hospital

Department of Otorhinolaryngology, 1P1

De Pintelaan 185

9000 Ghent

Belgium

Phone: 003293320864

Fax: 003293324993

E-mail: frederic.acke@ugent.be

**Additional Table 1: List of articles included in the meta-analysis.**

This table shows the articles from which data were extracted to use in the results section. For each article, the number of patients and families included, and the type of Stickler syndrome is mentioned, as well as some methodological features. References are numbered according to and can be found in the original article.

| **Article** | **Number of individual patients** | **Number of families** | **Stickler type** | **Methodology of obtaining audiological data and remarks** |
| --- | --- | --- | --- | --- |
| Admiraal (2000)[^17^](#_ENREF_17) | 15 | 1 | STL3 | Thorough audiological examination (showing the audiograms), also described in Vikkula et al. (1995)[^7^](#_ENREF_7) and van Beelen (2012)[^53^](#_ENREF_53) |
| Ahmad (1991)[^5^](#_ENREF_5) | 6 | 1 | STL1 | History taking |
| Ahmad (1995)[^18^](#_ENREF_18) | 6 | 1 | STL1 | History taking |
| Annunen (1999)[^19^](#_ENREF_19) | 12 | 12  (6 without individual data) | STL1/STL2 | Data from referring physicians, data from 1 family partly derived from Zlotogora et al. 1992 (Zlotogora J, Sagi M, Schuper A, Leiba H, Merin S. **Variability of Stickler syndrome.** *Am J Med Genet* 1992, **42**:337-9), 5 Stickler-like families |
| Avcin (2008)[^20^](#_ENREF_20) | 3 | 1 | STL3 | Audiological examination, also described in Li (2010)[^58^](#_ENREF_58) |
| Baijens (2004)[^21^](#_ENREF_21) | 2 | 1 | STL1 | Thorough audiological examination (showing the audiograms) |
| Baker (2011)[^9^](#_ENREF_9) | 8 | 1 | STL5 | Audiological examination in 2 patients, not specified in 6 patients |
| Ballo (1998)[^22^](#_ENREF_22) | 4 | 1 | STL1 | Audiological examination, Stickler-like family |
| Brown (1992)[^23^](#_ENREF_23) | 4 | 1 | STL1 | History taking and otorhinolaryngologic examination |
| De Keyzer (2011)[^24^](#_ENREF_24) | 7 | 1 | STL1 | Audiological examination in proband, not specified in family members |
| Donoso (2002)[^25^](#_ENREF_25) | 0 | 1 (without individual data) | STL1 | History taking and analysis of medical records |
| Edwards (2012)[^26^](#_ENREF_26) | 9 | 1 | STL1 | Not specified |
| Faber (2000)[^27^](#_ENREF_27) | 2 | 1 | STL1 | Not specified |
| Freddi (2000)[^28^](#_ENREF_28) | 5 | 1 | STL1 | Not specified |
| Fujiwaki (2004)[^29^](#_ENREF_29) | 1 | 1 | STL1 | Thorough audiological examination (showing the auditory brainstem response curves) |
| Gerth-Kahlert (2011)[^30^](#_ENREF_30) | 1 | 1 | STL1 | Audiological examination |
| Go (2003)[^31^](#_ENREF_31) | 13 | 1 | STL1 | Analysis of medical records, Stickler-like family (DRRD) |
| Hoornaert (2006)[^32^](#_ENREF_32) | 11 | 5 | STL1 | Analysis of medical records,1 family (2 patients) with brachydactyly |
| Hoornaert (2010)[^33^](#_ENREF_33) | 3 | 3 | STL1 | Data from referring physicians |
| Korkko (1993)[^34^](#_ENREF_34) | 3 | 1 | STL1 | Audiological examination in 1 proband, history taking in family members |
| Leung (2006)[^35^](#_ENREF_35) | 3 | 1 | STL1 | Not specified |
| Liberfarb (2003)[^11^](#_ENREF_11) | 33 | 8 | STL1 | Audiological examination in 23 patients, analysis of medical records in the others (reporting presence of sensorineural hearing loss), 6 families (21 patients) also described in Wilkin (2000)[^60^](#_ENREF_60), 5 families also described in Rose (2005)[^3^](#_ENREF_3) |
| MacRae (2006)[^36^](#_ENREF_36) | 1 | 1 | STL1 | Audiological examination |
| Majava (2007)[^37^](#_ENREF_37) | 6 | 6 | STL2 | Audiological examination, clinically rather Stickler syndrome than Marshall syndrome |
| McAlinden (2008)[^38^](#_ENREF_38) | 3 | 3 | STL1 | History taking |
| Mikhak (2006)[^39^](#_ENREF_39) | 2 | 1 | STL1 | Audiological examination |
| Nagendran (2012)[^40^](#_ENREF_40) | 4 | 3 | STL1 | Audiological examination, 2 patients with somatic mosaicism |
| Nikopoulos (2011)[^41^](#_ENREF_41) | 3 | 2 | STL4 | Thorough audiological examination (showing the audiograms) |
| Nishimura (2005)[^42^](#_ENREF_42) | 11 | 7 | STL1 | Data from referring physicians |
| Olavarrieta (2008)[^43^](#_ENREF_43) | 2 | 1 | STL1 | Thorough audiological examinations (showing the audiograms), patient with Stickler and BOR excluded |
| Parma (2002)[^44^](#_ENREF_44) | 0 | 1 (without individual data) | STL1 | History taking and medical records |
| Poulson (2004)[^45^](#_ENREF_45) | 25 | 6 | STL2 | Audiological examination and history taking, 1 family (6 patients) same as Richards (1996),[^6^](#_ENREF_6) 2 families (13 patients) same as Martin (1999)[^59^](#_ENREF_59) |
| Richards (2000a)[^46^](#_ENREF_46) | 11 | 3 | STL1 | Audiological examination |
| Richards (2006)[^47^](#_ENREF_47) | 16 | 3 | STL1 | Audiological examination |
| Richards (2000b)[^48^](#_ENREF_48) | 29 | 8 | STL1 | Audiological examination |
| Richards (2005)[^49^](#_ENREF_49) | 1 | 2 (1 without individual data) | STL1 | Audiological examination, 1 Stickler-like family (DRRD) |
| Ritvaniemi (1993)[^50^](#_ENREF_50) | 3 | 1 | STL1 | Not specified |
| Rose (2005)[^3^](#_ENREF_3) | 2 | 1 | STL1 | Audiological examination (reporting presence of high-frequency hearing loss), also described in Wilkin (2000)[^60^](#_ENREF_60) and Liberfarb (2003)[^11^](#_ENREF_11) |
| Sirko-Osadsa (1998)[^51^](#_ENREF_51) | 9 | 1 | STL3 | Audiological examination in 7 patients, history taking in 2 patients |
| Stevenson (2012)[^52^](#_ENREF_52) | 2 | 1 | STL1 | Audiological examination |
| van Beelen (2012)[^53^](#_ENREF_53) | 4 | 1 | STL3 | Thorough audiological examination (showing the audiograms) |
| Van Camp (2006)[^8^](#_ENREF_8) | 4 | 1 | STL4 | Thorough audiological examination (showing the audiograms) |
| Van Der Hout (2002)[^54^](#_ENREF_54) | 2 | 1 | STL1 | Not specified |
| Vuoristo (2004)[^55^](#_ENREF_55) | 3 | 1 | STL3 | Audiological examination in 2 patients, medical records in 1 patient |
| Yaguchi (2011)[^56^](#_ENREF_56) | 1 | 1 | STL1 | Not specified |
| Zechi-Ceide (2008)[^57^](#_ENREF_57) | 18 | 9 | STL1 | Not specified |
|  | **313** | **102 (+9 without individual data)** |  |  |
